# ‘Fix the issues at the coalface and mental wellbeing will be improved’: a framework analysis of frontline NHS staff experiences and use of health and wellbeing resources in a Scottish health board area during the COVID-19 pandemic

**DOI:** 10.1186/s12913-021-07103-x

**Published:** 2021-10-13

**Authors:** Catherine Clarissa, Sam Quinn, Rosie Stenhouse

**Affiliations:** Nursing Studies, School of Health in Social Science, University of Edinburgh, Elsie Inglis Quad, Teviot Place, EH8 9AG Edinburgh, United Kingdom

**Keywords:** COVID-19, Health facilities, Healthcare, Staff support, Staff health, Wellbeing, Employees

## Abstract

**Background:**

Frontline healthcare staff working in pandemics have been reported to experience mental health issues during the early and post-peak stages. To alleviate these problems, healthcare organisations have been providing support for their staff, including organisational, cognitive behavioural and physical and mental relaxation interventions. This paper reports the findings of a study commissioned by a Scottish NHS health board area during the initial outbreak of COVID-19. The study aimed to understand the experience of NHS staff relating to the provision of wellbeing interventions between March and August 2020.

**Methods:**

Data were gathered from free-text comments of eight surveys completed by a wide range of staff across sites within one NHS health board in Scotland. We conducted a framework analysis of the data.

**Results:**

Our findings show that despite the provision of relaxational and cognitive behavioural interventions to support staff wellbeing during the early months of the COVID-19 pandemic, there were barriers to access, including heavy workload, understaffing, inconvenient locations and the stigma of being judged. Organisational factors were the most frequently reported support need amongst frontline staff across sites.

**Conclusions:**

While relaxational and cognitive behavioural interventions were well received by staff, barriers to accessing them still existed. Staff support in the context of organisational factors, such as engagement with managers was deemed as the most important for staff wellbeing. Managers play a key role in everyday organisational processes and therefore are in the right position to meet increasing frontline staff demands due to the pandemic and removing barriers to accessing wellbeing support. Healthcare managers should be aware of organisational factors that might increase job demands and protect organisational resources that can promote wellbeing for frontline staff.

## Background

Since the World Health Organization (WHO) declared COVID-19 a global pandemic in March 2020 [1], health systems across the globe have experienced unprecedented pressure, particularly on healthcare staff working on the frontline [2–4]. Evidence from previous Severe Acute Respiratory Syndrome (SARS), Ebola and Middle East Respiratory Syndrome (MERS) pandemics identify both immediate and long term impacts on mental health [5–8]. Studies have found that the pandemic related risk factors for: post-traumatic stress disorder (PTSD), post-traumatic stress syndrome (PTSS) [9, 10], poor coping strategies [11], psychological distress and burnout [10] include a range of psychosocial [9, 11], organisational [9, 11] and individual factors [10].

Strategies for providing staff support during the COVID-19 pandemic focus either on the individual [12–14], team [13] or the organisation [13, 15]. Drawing literature from the Norwegian Institute of Public Health’s *Live map of COVID-19 evidence*, Muller et al. [13] conducted a rapid systematic review of the impact of the COVID-19 pandemic on healthcare staff’s mental health and implemented interventions. Of six studies which reported on interventions at organisational, team and individual level, none measured effectiveness or reported outcomes. There remains a dearth of evidence regarding the effectiveness of interventions designed to aid staff health and wellbeing during the COVID-19 pandemic. Approaches are therefore based on existing psychological and organisational theory.

The psychological approach focuses on the development of individual resilience by attending to individual self-care needs through interventions that promote relaxation, self-compassion and physical self-care [12–14]. Organisational approaches focus on organisational processes and resources to provide support such as encouraging social bonds between colleagues, and leaders performing end of shift reviews with staff [15] or re-structuring shifts and working weeks to support staff [13]. Organisations also need to meet basic staff needs for rest, information, the required safety equipment, appropriate shift patterns, and information about support resources [15]. Managers, therefore, become responsible for ensuring adequate preparation and support, provision of the required equipment to keep the workforce safe, and active monitoring and intervention where mental health deteriorates. Given the responsibility placed on managers, Greenberg et al. [16] identify the need for senior managers to support junior managers.

This paper draws on organisational theories relating to work-stress to support conceptual understanding of the data. Development of individual and organisational interventions can be understood from the perspective of the Job Demands-Resources (JD-R) theory of work-stress where staff wellbeing results from the interplay between the individual, job demands and job resources [17, 18]. Job demands are defined as ‘physical, psychological, social, or organisational aspects of the job that require sustained physical and/or psychological (cognitive and emotional) effort or skills’ [17], for instance, poor work environment, time and workload pressures, and emotional demands. Job resources relate to the aspects which motivate staff to achieve work goals and to meet job demands, such as autonomy, performance feedback, training, and professional development [17, 18].

The psychosocial safety climate (PSC) of an organisation reflects the value placed on the psychological health of workers by the management [19]. This PSC shapes job design and the social relations that are promoted within an organisation, both of which contribute to demands and resources experienced by workers [19]. Research has demonstrated that high levels of PSC are associated with high levels of worker psychological health, and low levels of PSC with poorer worker psychological health. Where espoused PSC is enacted through the provision of managerial support, Yulita et al. [20] found a positive relationship with worker psychological health, interpreting the enactment of the espoused PSC as indicating to workers that they were safe to take action to look after their psychological health (through engagement with resources to manage their work-stress).

This paper reports the findings of a framework analysis of data collected within a single NHS Board area in Scotland aiming to understand the experience of frontline staff accessing wellbeing interventions implemented during the initial outbreak of COVID-19 (March to August 2020). Within this NHS Board, a range of individual and organisational interventions were instigated across several sites to mitigate the impact of working during the pandemic (for examples see Table 1). These interventions were available to all employees, however, this analysis draws on data about the experience of frontline healthcare staff. We defined frontline staff as those staff who work in areas where they could come into contact with COVID-19 as part of their working practice. This included all staff within the acute hospitals, but excluded surveys of non-clinical support and administrative staff who primarily worked from home.

**Table 1:** Examples of the interventions implemented by the health board in response to COVID-19 pandemic

Organisational interventions
Facilities (Free parking, hot food available on nightshift, additional break spaces)
Communication (COVID briefing, move to virtual meetings)
Compliance with public health measures (Signage, social distancing, Perspex screens)
Organisation of clinical settings (Introduction of red and green areas, flexible work patterns)
Technology (Move to Microsoft Teams, virtual consultations, telephone triage)
**Physical and mental relaxation interventions**
Yoga and exercise
Massage and complementary therapies
Art therapy
Silent disco
Wellbeing hubs / rest areas
Wellbeing apps
Mindfulness and breathing awareness training
**Cognitive behavioural interventions**
Psychological and counselling services
Team check-ins/huddles
Telephone helplines
Self-care resources

### Research questions

Based upon queries emerging from reviewing current literature related to COVID-19 and the health and wellbeing experiences of healthcare staff working during pandemics, the project team developed the following research questions:

1. What were frontline healthcare staff experiences of working in one Scottish NHS Board during the early period of the COVID-19 outbreak (March-August 2020)?
2. In what ways did the interventions introduced during the pandemic meet staff wellbeing needs arising from working during COVID-19?
3. What were the barriers or facilitators to accessing wellbeing supports?

## Methods

### Design

A framework analytic approach was used to analyse secondary data gained from across the NHS Board. A framework approach was appropriate given the heterogeneity of the data [21], this approach enabled thematic development across the range of data.

### Data sources

The complete dataset primarily comprised qualitative data (free text comments) from eight different surveys carried out across a range of sites. Some surveys were open to all staff, whilst others were focused on professional groups such as doctors in training. Statements from a Black, Asian and Minority Ethnic (BAME) staff clinical network focus group were also included in the analysis. A full breakdown of the data sources is presented in Table 2.

The data had been collected by several different services using online survey methods with the aim of understanding staff experience during COVID-19. No standardised survey tool was used across the datasets. The questions asked across the data collection were phrased differently (for examples see Table 3). However, as the primary goal of each piece of work was to understand staff experience and support needs during COVID, there was sufficient convergence to create a coherent dataset. The data were provided to the research team by the NHS Board having been cleaned and anonymised. Most of this data took the form of Excel spreadsheets which identified each question and the responses.

**Table 2:** Breakdown of the data sources

Hospital	Data type	Participant professional group	Number of respondents (N=)	Response rate (%)
Acute Hospital 1	Wellbeing Programme Report	Respiratory/ ICU/ Pharmacy/ Admin	Not known	Not known
Acute Hospital 1	Survey	Not stated	128	3.3 %
Acute Hospital 2	Survey	Clinical/admin/ management	257	4.1 %
Acute Hospital 3	Survey	Not stated	162	5.9 %
Acute Hospital 3	Survey	Nurse/admin/ radiographer/estates/ medical/student/ Phlebotomy/ Pharmacist/ Electrician/ Community/ Charge	210	
NHS Board-wide	Survey	Doctors in Training (below consultant level)	151	12 %
NHS Board wide	Focus group	BME staff network (Clinical/admin/ non-clinical)	80	9.6 %
Mental Health services	Survey	Clinical/admin/ management	135	8.3 %

**Table 3:** Examples of survey questions

Hospital	Data type	Example Questions
Acute Hospital 1	Wellbeing Programme Report	What kind of emotional support do you envisage you and your staff will need over the coming weeks?
Acute Hospital 1	Survey	What would help with your wellbeing (at work) during this pandemic?
Acute Hospital 2	Survey	From a staff engagement and experience perspective, what has worked well over COVID-19? What could have been done differently?
Acute Hospital 3	Survey	In relation to the wellbeing of you and colleagues what has worked well? You may wish to consider wellbeing spaces, support services, helplines etc. In relation to the wellbeing of you and colleagues what could be better?
Acute Hospital 3	Survey	Was there anything in particular that was helpful about the session [resilience training]?
NHS Board-wide	Survey	List any positive changes you have noted from your [health board] as a result of the pandemic. What, if anything, do you think could have been done better?
NHS Board wide	Focus group	What has been the impact of Coronavirus for you? What would you want [health board] to do?
Mental Health services	Survey	What would help you with your wellbeing (at work) during this pandemic?

### Data analysis

Framework analysis was used to enable synthesis of the multiple sources of data [21]. Data were tabulated and sorted into key themes using five steps: familiarisation; identifying a thematic framework; indexing; charting; and mapping and interpretation [22, 23]. The familiarisation stage involved the research team (one academic and two post-doctoral research fellows – all with expertise in qualitative research) reading the free-text comments across datasets and discussing the key ideas from the data. Through this process, we noted the diversity of the support available across services, reflecting the two distinct approaches to providing wellbeing support; individual vs. organisational [12, 14, 15].

Recognising the broad range of interventions in our data, we developed a framework drawing on Ruotsalainen et al.’s [24] systematic review of 58 studies trialling interventions to prevent psychological stress in healthcare staff. Interventions were categorised as cognitive behavioural, relaxation, or organisational [24]. These three key categories guided the initial coding, with the framework further adapted throughout the analysis to reflect the emerging themes [25]. The adaptation included renaming ‘relaxation’ into ‘physical and mental relaxation’ to reflect specific aspects of the interventions (see Table 4).

**Table 4:** Categories and definitions of interventions (adapted from Ruotsalainen et al. [24]).

Category	Definition	Example
Cognitive behavioural	Interventions that aim to change how participants think, feel and consequently behave in stressful situations.	Telephone support line for staff, staff debrief
Physical and mental relaxation	Interventions that aim to induce a state of mental or bodily calmness, or both, to counteract the agitation caused by stress.	Yoga, mindfulness session, table tennis, silent disco
Organisational	Interventions that change resources, the working environment, work tasks or working methods.	Parking space, changing rooms, break areas, canteens

At the indexing stage, the data were coded into the framework categories in Microsoft Office 2016 Excel Spreadsheets. We added the ‘N/A’ category to classify vague or generic responses that were not referring to a specific intervention, for instance, ‘staff well-being support’ or ‘some workspaces didn’t work well’. We then collated the indexed data for each category and charted them under their corresponding themes. At the last stage of mapping and interpretation, we used our research questions as an analytic guide for understanding the data. We discussed our interpretation in regular meetings before finally developing a conceptual explanation of the data.

### Ethics

 Ethical review and approval for this project was gained from the research ethics committee in the School of Health in Social Science at the University of Edinburgh on 24th August 2020.

## Results

The response rate for the hospital surveys ranged from 3.3 to 8.3 %. Despite low response rates the qualitative data on which the findings of this paper are based demonstrated saturation of responses. We can hypothesise that the surveys that yielded better response rates did so because they were targeted at specific sub-populations and were therefore perhaps experienced as more relevant.

Findings are presented under the headings of organisational factors; physical and mental relaxational interventions; and cognitive behavioural interventions. Our results suggest that there was greater uptake of relaxational interventions than cognitive behavioural ones. While a range of relaxational and cognitive behavioural interventions was covered, staff responses most frequently related to organisational factors, which is reflected in the balance of the subsequent discussion. Responses point to respondents’ recognition that it is organisational systems and practices that most impact staff wellbeing and that these, rather than short term individual interventions, should be the focus of ongoing development: *‘It’s lovely to have staff zoom yoga but we would prefer to have better food in the canteen, get our rotas earlier, feel consistently supported by senior staff and management… Let’s try and engage with proper culture change not just pick the low hanging fruit’* (Doctors in Training).

### Organisational factors

This theme describes how a range of material and procedural facets of the organisational context supported participants or were identified as gaps in the support provided. Across this theme staff identified organisational issues that pre-dated the COVID-19 pandemic which could be, or had been temporarily, ‘fixed’ to provide lasting opportunity for improved mental wellbeing: ‘*Fix the issues at the coalface and mental wellbeing will be improved’*.

*Facilities.* Many participants commented that the introduction of basic amenities such as free parking, hot food and drink availability during the night shift, and the provision of spaces that staff could go to take a break was supportive. Many participants identified the need for space to reflect, eat, meet with colleagues socially at break times away from the public and patients. There was also an identified need for changing facilities.

*‘Staff rest areas, staff recreation areas, staff coffee/lunch areas separate to the public so staff can relax and debrief. Sadly lacking until now.’* (Acute Hospital 1).

The provision of specific wellbeing areas for relaxation, alongside the provision of other on-site or online support services, were appreciated.

*‘Staff wellbeing should remain a priority. The difference it makes in people’s attitudes when they feel valued and looked after is immense. This then allows us to look after our patients better.’* (Acute Hospital 2).

These data demonstrate the positive change in participants’ experience of the organisation as it started to demonstrate that it valued the wellbeing of its staff through providing for their needs. However, support for the wellbeing facilities was not unanimous, with some feeling these wasted the limited NHS budget.

*Clinical area organisation.* Many clinical staff participants felt the organisation of clinical areas with designated zones and improved staffing rotas for medics worked well. However, some clerical and administrative staff attached to clinical areas felt that their needs were not considered.

*‘… it is terrible that reception staff in outpatient areas do not have Perspex screens to protect them. You walk in anywhere else and there are screens everywhere.’* (Acute Hospital 3).

There were reports of both over and understaffing of wards, with some respondents questioning if the workforce had been deployed to a maximum effect during the pandemic. Many staff were redeployed to unfamiliar areas with the perceived lack of preparation provided to some creating increased stress.

*‘Some staff that have been redeployed to unfamiliar areas have had poor preparation and support in knowledge and skills relevant to the area. Some staff have felt at times, unsafe in their practice with a feeling of putting both their professional registration and patient care needs at risk.’* (Acute Hospital 2).

Staff in understaffed areas experienced increased stress and found they were unable to engage with wellbeing initiatives due to workload pressures. Conversely, overstaffing led to greater demand for the available health and wellbeing interventions.

*Teamworking.* When asked what the most positive outcome from the pandemic was, staff identified teams working more closely together with a common goal. This was experienced as increased collegiality or ‘camaraderie’ and a sense of ‘being in it together’ with comments noting increased consideration of colleagues and their wellbeing, and kindness towards each other.

*‘The commitment, efforts, passion, courage, teamwork and so on, needs to be celebrated and marked in some way that means something to the staff’* (Acute Hospital 2).

However, this camaraderie was not universally experienced with many BAME healthcare staff feeling unprotected and disempowered.

*‘Each BAME death terrifies me. It does not seem like my non-BAME counterparts understand the severity or fear we have. They are happy to donate more clinical work to me than they are to take on themselves. A peer of the same standing would quibble about this and so are not given this work, and they know I am likely to do the work without resistance.’* (BAME Network).

Some participants identified incivility, including bullying, blaming, discrimination and harassment. Participants from the BAME network reported an increase in racism towards colleagues of East Asian heritage. The survey data also indicated that the culture in some areas was a barrier to supportive teamwork.

*Engagement of managers.* Across the hospital-wide surveys, staff expressed a desire for managers who were open, compassionate, able to listen to and show interest in staff work and issues, particularly around their health and wellbeing.

*‘It would have been good if the more senior managers had made an effort to visit areas … and acknowledge the work being done by their staff….* [and] *acknowledge bereavements…’* (Acute Hospital 3).

Respondents felt disconnected from senior management and requested more opportunities to engage with managers through direct encounters, and discussion rather than unidirectional communications.

*‘*[I would have liked] *to be involved in discussions around potential measures before they are imposed (if time allows), and for the management at all levels to allow a genuine exchange of views.’ *(Mental Health Services)*. *

Participants across the datasets wanted recognition of their contribution, and that of non-clinical staff, by managers.

*‘We focus a lot on medical and nursing but I think there needs to be more and better recognition for other services on-site: management, admin, domestics, porters etc. All the work we do feels unvalued’* (Acute Hospital 1).

*Communication.* Communication was a key focus across the surveys with some participants identifying examples of good strategies for ‘top down’ communication such as daily all-staff bulletins, huddles, bulletins, daily emails, and corporate communications. These forms of communication enabled participants to remain informed of rapid changes in organisational policy and practice. Most comments identified problems with communications. Many participants wanted clearer, more timely communication.

*‘Better communication of NHS plans at the start of the pandemic. I appreciate plans were changing and the situation was dynamic but felt trainees weren’t part of the decision-making process or listened to when we could have contributed’*. (Doctors in Training)

Some staff groups felt excluded from some of the communication points such as huddles, or that their voices were not heard. Many of the staff who felt that they were not heard were non-clinical staff such as laboratory services staff who asked that hospital managers ‘*not so much continue to prioritise as start to try to listen/engage with staff engaged in performing the practical work …’* (Acute Hospital 2). There was a recognition by some respondents that staff such as domestics and porters had little voice; interestingly these groups are not identified in the respondent groups in Tables 2, perhaps lending support to this point.

BAME staff, a group identified as at increased risk of serious illness and death from COVID-19 [26], also felt unheard. The specific increased risk from infection experienced by this group of respondents increased the sense of urgency that managers hear and respond to their concerns:

‘M*anagers have a role in identifying and listening to BAME staff…. to ask BAME staff about their concerns, this may help those who do not or cannot voice their concerns to be given the opportunity to … speak up and be listened to’* (BAME network).

There was detectable anger from some respondents who experienced a lack of ‘honesty’ of some of the organisational communication in relation to the level of risk posed to them, leaving them feeling vulnerable and unprotected:

*‘Be honest about risks. Be honest about if masks are not offering any protection or only slowing the risk of spread?? (sic) We are almost being thrown to the wolves and left to fend for ourselves!’* (Acute Hospital 3).

### Physical and mental relaxation interventions

Physical and mental relaxation interventions are designed to reduce stressful thoughts and facilitate mental and physical calmness [24]. The most well-received relaxation interventions had aspects of socialising, peer support and access to an area for rest.

*Physical relaxation interventions.* Yoga and massage were the two physical interventions that participants identified using most. Data indicated significantly less engagement with complementary therapies and art therapy.

*Mental relaxation interventions.* The most widely used mental relaxation interventions were wellbeing hubs and rest areas. A persistent theme explaining the popularity of the hub was the feeling of a safe space and separation from work areas.

*‘Being able to access quiet space and time to switch off - even for 15 mins.’* (Mental Health Services).

*‘I have…referred staff to the Wellbeing hub, most staff just for a break away to reflect and have quiet time.’* (Acute Hospital 3).

Wellbeing hubs were identified by many participants as something they hoped would be extended beyond the pandemic.

*‘Looking to the future after the pandemic, what would help with your wellbeing, here at the [hospital]? For the wellbeing hub to remain so staff can go and relax out of sight of public and patients.’* (Acute Hospital 1).

Few participants reported engagement with wellbeing apps like Headspace. Some staff proposed virtual relaxation training as an alternative that would allow them to interact with colleagues.

### Cognitive behavioural interventions

Cognitive behavioural interventions aim to give the user strategies to reflect on their thoughts and manage stress and negative emotions [27]. The findings of the framework analysis suggest that these types of intervention were accessed least across services. The cognitive behavioural interventions that staff identified as most beneficial for health and wellbeing were psychological and counselling services, and team huddles with colleagues. Telephone helplines and self-care resources were usually perceived as something positive to have access to but generally had low uptake.

*‘The* [NHS Board] *Occupational Health Clinical Service … rose admirably to the challenge of a phenomenally increased workload and were proactive in making efforts to support individuals’* (Acute Hospital 2).

*‘Telephone support line for staff was well-intentioned but low uptake reflects the fact that face-to-face support, ideally from a familiar face, is much more effective’* (Acute Hospital 2).

### Barriers to accessing support

*Organisational.* In areas where relaxational and cognitive behavioural interventions had low uptake, the reasons given for not accessing interventions were largely organisational factors. Key barriers to accessing wellbeing support included heavy workload, understaffing, inconvenient locations and not having sufficient time; *‘ too busy upskilling to be ITU Nurse* [to be able to access interventions].*’* (Acute Hospital 1).

*‘… there are a number of wellbeing services but don’t feel they are very accessible. [We need to be] addressing understaffing and lack of equipment required to cope with workload… wellbeing should be a lasting priority.’* (Acute Hospital 3).

Suggestions given to improve accessibility include *‘some of the services being delivered in ward areas, when safe to do so’* (Acute Hospital 1), and greater flexibility, particularly around the length of staff breaks, to accommodate access; ‘*not be tied to solely your 30-minute lunch break.’* (Acute Hospital 3).

*Location.* Some respondents identified that the lack of proximity of interventions to their place of work was a barrier to access.

*‘Lots of access to wellbeing materials, however, my main site is not in the hospital. I work in the community so didn’t have the same access, felt less valued than hospital staff.’* (Acute Hospital 3).

*Stigma.* A minority of participants who had not used any services indicated a sense of fear of being judged.

*‘Haven’t used any. I’m in the management department it just doesn’t seem appropriate to utilise the services.’* (Acute Hospital 1).

*‘Helplines have been available however I did not want my boss thinking I could not manage therefore did not access groups or support when on reflection I may have benefited from some 1:1 support’* (Acute Hospital 3).

*It’s not for us.* Another comment made by staff from the laboratory department indicates their hesitation to access the available services:

*‘I know a lot of first come first served hospital-wide stuff has been put on but laboratory staff are so insular they always think it’s not meant for them. If we had a regular session once a week only for our staff in our workplace it would encourage uptake and help with bonding/relaxation.’* (Acute Hospital 3).

*Individual preference.* There appeared to be a tension between approaches used in delivering the interventions and individual staff preferences. For example, whether break rooms should be used for quiet breaks or as a socialising space, some preferring individual interventions while others preferred group activities.

*‘Sadly I haven’t accessed them [activities]. Saw staff through the window in small groups and felt silly potentially going in on my own.’* (Acute Hospital 1).

Others felt they did not require the support.

*‘Honestly not had time to do any of these things while at work, and in my own time I find it more relaxing to leave and be with my partner’* (Doctors in Training).

The availability and variability of wellbeing support for staff were appreciated by many respondents across the data. A variety of resources were deemed useful in accommodating staff different needs.

*‘I think the resources that have been introduced are great for helping our wellbeing. Everyone has different needs when it comes to wellbeing so it would be difficult to introduce resources to suit everyone.’* (Acute Hospital 2).

## Discussion

During this early period of the COVID-19 pandemic frontline staff were thrown into a situation with high physical, psychological, cognitive and emotional demands on them as they developed new ways of working and navigated the rapidly changing clinical environment. The organisation responded by providing a range of organisational and individually focused resources.

In the face of increased job demands, much of the support desired by participants related to organisational processes and structures. Participants identified the importance of clear, concise and consistent communication between and across layers of the organisation to enable them to deal with the evolving protocols and policies. The importance of managers engaging with staff and taking account of their experiences in decision making, or ensuring adequate training for those staff who were redeployed, are again features of the everyday fabric of the organisation. While the additional services focused on developing personal resilience were appreciated, staff were clearly looking towards effective use of everyday organisational processes as a means of managing the demands of the pandemic. Such a focus from participants highlights the importance of organisations, and by implication, managers, focusing on how they can shape everyday processes to meet increasing demand quickly.

As the group of individuals within the organisation who have the power to effect change, managers become a focus for staff in relation to the implementation of organisational practices and support structures. The data contains many references to expectations and experiences of the relation between staff and managers focused on being listened to, engaged with or acknowledged by managers (usually those beyond direct line managers). Being heard, or having a voice, within the organisation is understood as a key mechanism through which workers can influence management’s actions. Whilst having a voice can take the form of direct discussion, surveys such as those being analysed here can be understood as a means of enabling staff to have a voice within the organisation. The notion of voice within organisations is nevertheless complex as it is bound up in the complexity of organisational hierarchies and power structures which permeate organisations from the wider social structures of society placing members of social groups in relation to one another [28]. Thus, not everyone has equal access to use their voice or be heard. It is important to consider how power might prevent staff from having a voice, because feeling able to influence what happens in the workplace has been found to be associated with feeling valued, increased sense of engagement, and staff retention [29, 30]. The desire or expectation of engagement with managers is perhaps indicative of the connection that this has with feelings of psychological safety and how opportunities for engagement with managers influences PSC of an organisation.

It follows then that those staff who feel they do not have a voice within the organisation, feel disenfranchised, and devalued. This was particularly evident in the data surrounding the experience of BAME staff who felt that their concerns were not heard by either other staff, or more particularly by managers who had the power to instigate changes to enable them to feel safer. However, a range of respondents including doctors in training and laboratory staff also identified a feeling of not being heard within the management structures. Where groups feel unheard, and therefore unsafe, this impacts their perception of the organisation’s PSC and may have a detrimental impact on wellbeing as has been found by some studies where lower PSC was associated with burnout and poorer wellbeing [31–33].

In addition to the lack of support experienced by those who felt unheard, those who experienced structural or geographical barriers to accessing the individual supports on offer felt unsupported. Data indicated that these barriers to accessing the support led to frustration from staff and potentially a sense that they were less valued by the organisation although this is not directly referenced. Whilst the authors acknowledge the difficulties of implementing individual support interventions so that they were geographically accessible to all, the potential impact on staff’s sense of being cared for or valued by their organisation is useful to understand as it has consequences for retention and staff wellbeing [30, 34].

This discussion has focused on the findings that organisational factors, material and relational, were central to the staff’s experience of working during the initial months of the COVID-19 pandemic. The findings highlight the material needs of the staff for resources and processes to help them manage their work. As key players within organisations, managers’ understanding of the sources of increased job demands, and resources that will enable staff to deal with these, is critical to the prevention of burnout [35, 36]. However, we found that the provision of these was bound up in participants’ experience of their relation with the organisation and management, influencing their sense of safety. That it is such aspects of the everyday fabric of the organisation are implicated in staff experience of support and psychosocial safety can inform management action in health service organisations. This is important because the perception that the organisation cares can support motivation and ongoing effort [37] during the peak of the pandemic when the demands on staff were high. High PSC has also been found to act as a buffer to fatigue [37] and enable staff to take action to look after their health [20].

The finding that it is everyday processes and structures that staff feel can provide the necessary resources to manage the job demands is important to support managers to fulfil this need. Whilst it is possible that being seen to provide some support, and therefore valuing staff wellbeing, has some impact on wellbeing [19], there is a need for managers to provide the right support in the right place at the right time to prevent burnout [15].

### Implications for research policy and practice

#### International relevance

The COVID-19 pandemic has created challenges for healthcare services across the world, with no service immune to the challenges of working throughout the pandemic and the threat posed to staff health and wellbeing. In areas with less well-resourced health systems, the potential impact is even greater, with evidence to suggest that COVID-19 is disproportionally affecting people living in poorer areas, minorities and a broad range of vulnerable populations [38]. Nevertheless, one of the notable findings of this study is the extent to which organisational factors plays a role in staff health and wellbeing. While physical infrastructure will always be important and remain a pertinent challenge for less resource rich areas, our findings suggest that management have a substantial part to play in supporting staff health and wellbeing through the cultural tone they set. In this sense, our findings can be used to inform the development of staff support, especially from the management, during future outbreaks of COVID-19 and other health crises across global healthcare services.

## Strengths and limitations

A key strength of this study was the use of the qualitative approach to analysis and data collection, where the use of free text questions enabled staff to focus on what was important to them, enabling them to provide responses that move beyond their experience of the specific interventions that had been implemented in the health board to identify a range of organisational issues. The heterogeneity of the data, gathered from a range of staff via a range of qualitative survey questions at different time points provided a rich dataset. The strength of the analysis is demonstrated through the congruence and consistency of themes identified across the data. Across the hospitals and services where data collection was undertaken, a substantial range of interventions was implemented. Feedback on these interventions provides valuable insight for health managers with a responsibility to support staff health and wellbeing.

We recognise that the datasets were limited by the lack of some staff perspectives, such as those of community and service staff. The impact of COVID-19 on the wellbeing of these groups remains a vital area for future research. Secondly, there was variance in the phrasing of the questions asked across the data collection which limits direct comparability of findings between groups of respondents or hospital sites. However, all questions focused on the main aim of the analysis which was to develop an understanding of staff experience.

## Conclusion and recommendations

The participants in this study strongly valued the provision of support during the COVID-19 pandemic, interpreting this as demonstrating that the organisation valued their wellbeing. While the organisation implemented individual relaxation and cognitive behavioural interventions, the most sought after support was that provided through everyday organisational structures and processes, and engagement with managers. It seems clear that the organisation needs to understand the perceptions and needs of their staff and build on this understanding to retain the increased level of PSC that was mostly experienced by staff. In this way, they reduce the risk of loss of motivation and engagement, burnout and diminished staff wellbeing following the pandemic when undoubtedly the additional individual interventions will be scaled back or removed.

### Authors’ information

*Catherine Clarissa* is a teaching fellow at the University of Edinburgh, Nursing Studies, School of Health in Social Science, Edinburgh, United Kingdom.

*Sam Quinn* is a research fellow at the University of Edinburgh, School of Health in Social Science, Edinburgh, United Kingdom. 

*Rosie Stenhouse* is Senior Lecturer, Nursing Studies, and Associate Director, Centre for Creative-Relational Inquiry, School of Health in Social Science, University of Edinburgh, Edinburgh, United Kingdom.

## Data Availability

The datasets analysed during the current study are not publicly available due to the anonymity requirements of the ethical permissions granted for this study, but are available from the corresponding author on reasonable request and with permission of NHS Board Executive Nurse Director.

## Abbreviations

BAME: Black, Asian and Minority Ethnic
COVID-19: Disease caused by SARS-CoV-2
JD-R: Job Demands-Resources
MERS: Middle East Respiratory Syndrome
NHS: National Health Service
PSC: Psychosocial Safety Climate
PTSD: Post-Traumatic Stress Disorder
PTSS: Post-Traumatic Stress Syndrome
SARS: Severe Acute Respiratory Syndrome
WHO: World Health Organization
